# Identification of a *β*-Arrestin 2 Mutation Related to Autism by Whole-Exome Sequencing

**DOI:** 10.1155/2020/8872577

**Published:** 2020-11-04

**Authors:** Yunfei Tang, Yamei Liu, Lei Tong, Shini Feng, Dongshu Du, Fuxue Chen

**Affiliations:** School of Life Sciences, Shanghai University, Shanghai 200444, China

## Abstract

Autism spectrum disorder (ASD) is a complex neurological disease characterized by impaired social communication and interaction skills, rigid behavior, decreased interest, and repetitive activities. The disease has a high degree of genetic heterogeneity, and the genetic cause of ASD in many autistic individuals is currently unclear. In this study, we report a patient with ASD whose clinical features included social interaction disorder, communication disorder, and repetitive behavior. We examined the patient's genetic variation using whole-exome sequencing technology and found new de novo mutations. After analysis and evaluation, ARRB2 was identified as a candidate gene. To study the potential contribution of the ARRB2 gene to the human brain development and function, we first evaluated the expression profile of this gene in different brain regions and developmental stages. Then, we used weighted gene coexpression network analysis to analyze the associations between ARRB2 and ASD risk genes. Additionally, the spatial conformation and stability of the ARRB2 wild type and mutant proteins were examined by simulations. Then, we further established a mouse model of ASD. The results showed abnormal ARRB2 expression in the mouse ASD model. Our study showed that ARRB2 may be a risk gene for ASD, but the contribution of de novo ARRB2 mutations to ASD is unclear. This information will provide references for the etiology of ASD and aid in the mechanism-based drug development and treatment.

## 1. Introduction

Autism spectrum disorder (ASD) is a complex, lifelong, neurodevelopmental disease with a high degree of genetic heterogeneity, characterized by impaired social communication and interaction skills, stereotyped behavior, declining interest, and repetitive activities [1, 2]. The prevalence of ASD has been steadily increasing since the first epidemiological study, and seven studies have shown that 4.1 of every 10,000 people in the UK have ASD [3]. Early research indicates that the incidence of ASD in men is 4–5 times that in women, although this difference is reduced among individuals with intellectual disabilities [4]. Genetic factors play a key role in the etiology of ASD and are combined with early environmental factors. Studies have shown that the incidence of childhood trauma, self-mutilation, and suicidal behaviors and thoughts of ASD patients is significantly increased [5], resulting in a heavy financial burden on families and society [6]. In the UK, the lifetime cost of ASD patients is £920,000 (US$1.36 million) [7].

Recent studies have shown that, although the heritability of ASD is high, the disease has a high degree of genetic heterogeneity, with rare and common genetic mutations [8–10]. Furthermore, the genetic etiology of approximately 90% of ASD patients is not clear [11, 12]. Common genetic variants have a weak effect on the risk of ASD. The combined effect of common low-impact genetic variants is also related to ASD. Rare variants that affect the risk of ASD include hundreds of genes in total [13, 14]. Genetic and epidemiological studies show that de novo mutations, that is, spontaneous rare mutations that do not occur in unaffected parents but occur in affected children [15, 16], contribute significantly to ASD, and approximately 3%–10% of de novo mutations in ASD-related risk exons are explained [9, 17, 18].

In this study, we examined a family including a child with ASD and found a new de novo mutation of ARRB2. Gene expression experiments confirmed the possible connection between ARRB2 and ASD. It is important to analyze mutations of a single disease-causing gene in ASD to understand its mechanism and physiological effects.

## 2. Materials and Methods

### 2.1. Patients and Samples

We obtained the medical history of a child with ASD. The patient's language comprehension was severely impaired, the tone and speed of speech were abnormal, the patient failed to establish relationships with other children and experienced extreme loneliness, and the patient's posture was rigid with repetitive movements. To further investigate the cause of ASD, we collected peripheral blood from the patient and the patient's parents and obtained the gDNA of three family members. Additionally, the family history of the proband was collected. All participants signed an informed consent form.

### 2.2. Whole Exome Sequencing

Whole-exome sequencing (WES) was completed at the Shanghai Yuanshen Biomedical Technology Co., Ltd. The company used the Agilent's liquid-phase chip capture system to efficiently enrich human exon region DNA and then performed high-throughput and deep sequencing on the NovaSeq 6000 platform. An Agilent SureSelect v6 kit was used to build the library and analyze data. The library was qualified, and the NovaSeq 6000 platform was used for sequencing according to the effective concentration of the genes in the library and the data output requirements. The final sequencing results were exported in Excel format. After obtaining the original sequencing reads, the reference genome (hg38) was used for bioinformatic analysis.

### 2.3. Analysis of Sequencing Results

#### 2.3.1. Evaluation of Mutation Sites and Screening of Candidate Genes

Online software programs including LRT, MutationTaster, MutationAssessor, and VEST3 (https://sites.google.com/site/revelgenomics/) were used to predict the pathogenicity of the mutations, and the GERP, phyloP, and SiPhy programs were used to analyze the conservation of mutation sites [19]. Additionally, using the Gene Ontology (GO, http://www.geneontology.org/) database, genes were classified according to their roles in biological processes, cell components, and molecular functions, and the Discovery Studio18 was used to simulate the wild type and mutant site protein structure.

#### 2.3.2. Gene Coexpression and Genetic Interaction Network

We extracted a dataset of ASD genes and performed weighted gene coexpression network analysis (WGCNA) [19]. This data set comprised a population of boys with relatively spontaneous ASD and excluded patients with known genetic diseases or recognizable phenotypes or syndromes, as well as patients with mental retardation or primary seizures. Obvious expression characteristics were found between individuals with ASD and sibling controls. This data was the chip data, removed one-to-many probes and selected high-expressed many-to-one probes.

A variety of relevant action modes provided by the STRING database were selected, mapping was performed, which indicated different action modes, and weighted values were provided by the database.

### 2.4. Animals

All animal experiments performed in this study were approved by the Animal Protection and Utilization Committee (Department of Experimental Animal Science, Shanghai University) and were strictly performed in accordance with the Animal Care and Used Program of the Experimental Animal Institute of Shanghai University.

Both C57 males and females aged 3–4 weeks were obtained from the Shanghai Experimental Animal Center, Chinese Academy of Sciences. All mice were reared under standard conditions (23 ± 2°C; 55% ± 5% humidity) on a 12-hour light-dark cycle.

Adult male and female mice were mated overnight. The date of detection of a vaginal plug was recorded as day 0.5 of pregnancy. Adult female mice were given a single intraperitoneal injection of valproic acid (VPA) (500 mg/kg in 0.85% saline) on day 12.5 of pregnancy. The control group was injected with the same dose of normal saline at the same time. Female mice were allowed to breed freely, and mice were weaned on day 23 after birth. Subsequently, male mice were selected as experimental subjects. Behavioral tests were performed on mice at 7–8 weeks.

### 2.5. Behavioral Tests

Behavioral tests were performed on mice according to the method described by St Omer et al. [20]. At least 10 experimental animals were included in each group. See the Supplementary Materials 2.5.1-2.5.4 for test methods.

### 2.6. Western Blotting

In western blot experiments, a ProteinExt mammalian membrane protein extraction kit (Transgen Biotech) was used to extract proteins from tissue samples of the hippocampus in C57 mice, and a BCA kit (Beyotime Shanghai, China) was used to determine the protein concentration in the tissue. Thirty microliters of protein from each sample was separated on a precast gel (Tanon, Biofuraw), and the protein was transferred to a polyvinylidene fluoride membrane (Immobulon-P 0.45 mm, Millipore Germany) using transfer solution. The transferred membrane was blocked with QuickBlockTM Western blocking buffer (Beyotime, P0252) for 30 minutes at room temperature and incubated with the primary antibody at 4°C overnight (rabbit anti-*β*-Arrestin2 (CST, 1 : 1000) or mouse anti-GAPDH (Affinity: 1 : 3000)). After washing with phosphate buffered saline with Tween (PBST), the membrane was incubated with a horseradish peroxidase-linked anti-rabbit or anti-mouse secondary antibody (1 : 10,000 Santa Cruz Biotechnology USA) in PBST with 5% BSA at room temperature for 1 hour. Immunoblots were visualized using ECL (Immobulon, Millipore, Germany) and Image Develop (Tanon5200S China).

### 2.7. Immunofluorescence

First, slides containing the cells were immersed three times in a petri dish containing PBS. Next, the slides were fixed with 4% paraformaldehyde for 15 minutes and incubated with 0.5% Triton X-100 (prepared in PBS) for 20 minutes at room temperature. The slides were blocked at room temperature for 30 minutes. Then, the blocking solution was drained, a sufficient amount of the diluted primary antibody (rabbit anti-*β*-Arrestin2 (PTG, 1 : 200) or mouse anti-Neun (Abcam: 1 : 500)) was added, and the slides were placed in a wet box and incubated at 4°C overnight. Next, the slides were dipped in PBST, the excess liquid on the slides was drained, and a diluted fluorescent secondary antibody (Alexa Fluor 594 anti-rabbit (1 : 500; Abcam) or Alexa Fluor 488 anti-mouse (1 : 500; Abcam)) was added. The slides were protected from light and incubated for 1 hour at 20–37°C in a wet box. Afterwards, the slides were incubated for 5 minutes in 4′,6-diamidino-2-phenylindole (DAPI) and protected from light. The specimens were stained, excess DAPI was washed off with PBST, liquid was blotted from the slides, mounting solution containing an antifluorescent quencher was applied, and the slides were observed with a confocal laser scanning microscope (LSM 710 Carl Zeiss).

### 2.8. Statistical Analysis

Data are expressed as the mean ± SEM. A one-way variance method was used, and statistical analysis was performed using Origin 8, followed by a post hoc minimum significance test. A value of *P* < 0.05 was considered statistically significant.

## 3. Results

### 3.1. Analysis of Sequencing Results and Candidate Gene Selection

Blood samples were collected from child with ASD and their parents. The quality control of WES analysis is described in Table 1 in the Supplementary Materials. Many mutations were detected in the analysis, but the number of mutations associated with the disease was limited. To screen out mutations that were related to the disease from the large number of mutations detected, we used existing databases, software, and other tools, including the 1000 Genomes, EXAC, and esp6500siv2_all databases, to screen these mutations based on genes, mutation types, mutation details, population mutation frequency, and pedigree spectrum. The 1000 Genomes, EXAC, and esp6500siv2_all databases were searched for common mutations in the population while excluding rare mutations (< 0.05). The mutations that were present in the patient but not in the parents were determined based on a family map. After preliminary screening, 20 insertion-deletion (INDEL) (Supplementary Materials Table 2-1) and 88 single nucleotide polymorphism (SNP) sites (Supplementary Materials Table 2-2) were obtained. Next, the LRT, MutationTaster, MutationAssessor, and VEST3 tools were used to predict the harmfulness of all mutation sites. Additionally, the conservation of mutation sites was analyzed using the GERP, phyloP, and SiPhy tools. The sites that were more conserved had a greater impact on the protein. Based on the above prediction results, we selected five candidate genes (Table 1). Next, the GO database was used to classify the mutant genes according to their roles in biological processes, cell components, and molecular functions (Figure S1). We screened three GO annotations related to synapses (Table 2, [21]. Combined with the previous analysis of the harmfulness of conserved mutation sites, the target gene ARRB2 was identified.

### 3.2. Distribution of the Candidate Gene

**Figure 1:**
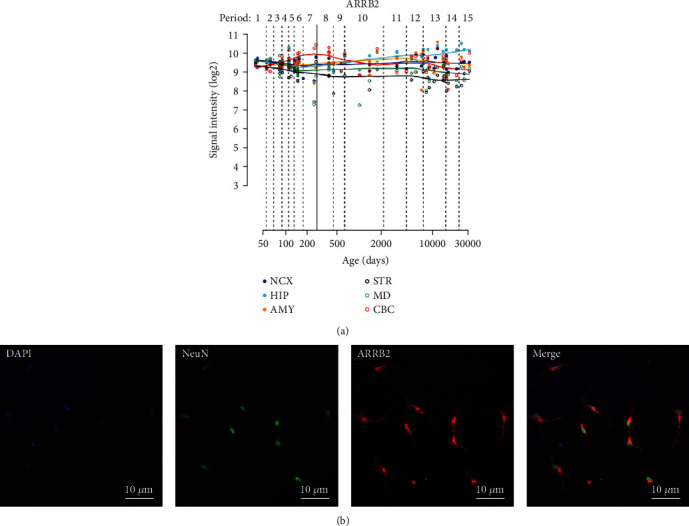
Distribution of ARRB2. (a) The expression levels of ARRB2 are shown throughout the entire development and adulthood. (b) Colocalization of Neun (green) and ARRB2 (red) in the neuron.

Subsequently, the expression of ARRB2 in different brain regions and developmental stages was evaluated to investigate the potential contribution of this gene to the human brain development and function. We obtained gene expression levels measured using microarrays from the human brain transcriptome database (HBT, http://hbatlas.org/) [19]. Throughout development and adulthood, ARRB2 is stably and highly expressed in the brain regions of the neocortex, hippocampus, amygdala, striatum, mid-thalamic nucleus, and cerebellar cortex. (Figure 1(a)). Next, we extracted primary neurons from the hippocampus of newborn C57 mice for immunostaining. Blue fluorescence indicated DAPI-stained nuclei, red fluorescence indicated cells expressing ARRB2, and green fluorescence indicated Neun-positive cells. The results showed that ARRB2 was expressed in the hippocampus and was colocalized with neurons (Figure 1(b)).

### 3.3. Coexpression and Interaction of ARRB2 and ASD

**Figure 2:**
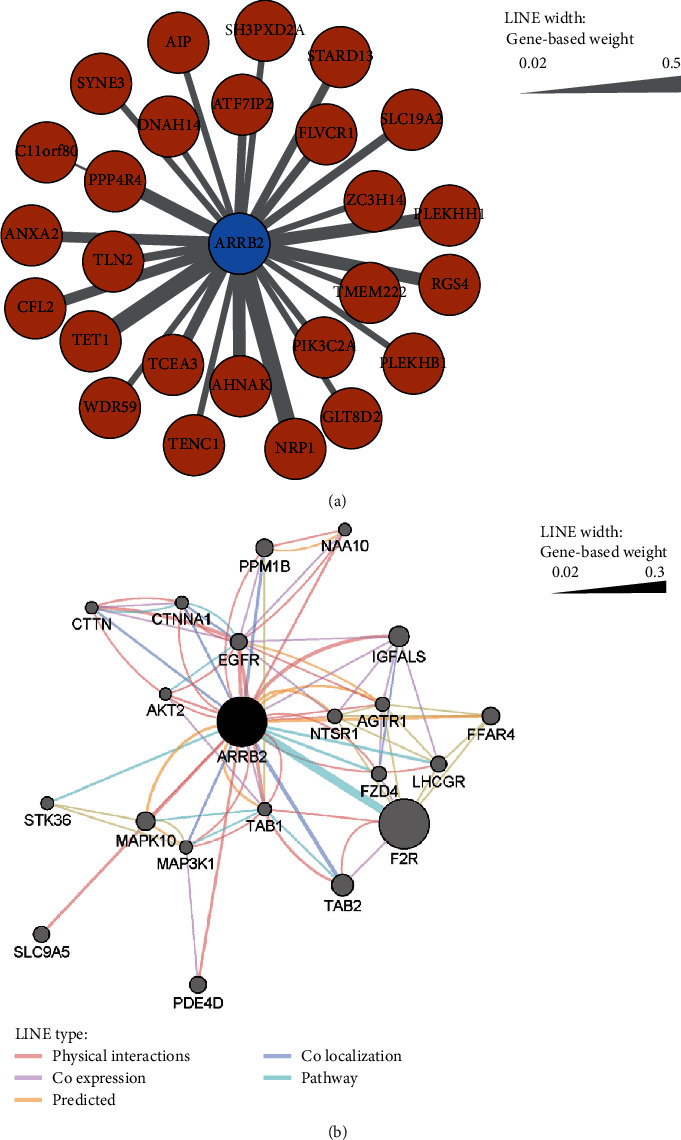
(a) Coexpression network analysis of ARRB2. (b) Analysis of the genetic interaction network of ARRB2. A larger weight value indicates a greater correlation.

We used the GEO dataset GSE65106, which includes 21 samples from ASD patients. A total of 21,408 pointers were analyzed, with 21,408 corresponding genes. WGCNA was used to analyze the coexpression network. The genes that were associated with ARRB2 were selected from the coexpression network and clustered into a module by WGCNA. A total of 26 related genes were selected. A network diagram was constructed based on its topological overlap measure (TOM) correlation with ARRB2 (Figure 2(a)). Additionally, a variety of related action modes provided in the STRING database were selected, and a graph was created to show the different action modes and the weight value provided by the database (Figure 2(b)). A larger weight value indicated a greater correlation.

### 3.4. Mutation of ARRB2

**Figure 3:**
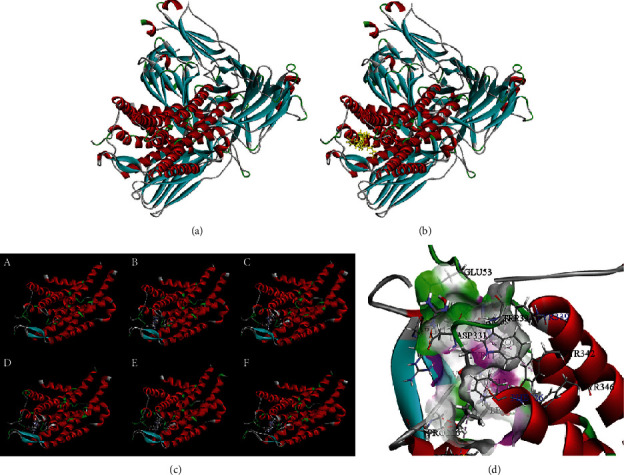
(a) Structure of wild type ARRB2. (b) Structure of wild type ARRB2 binding ligand. (c) Stable structure of wild type ARRB2 binding to the ligand. (d) Binding site in the active pocket.

By combining the LRT, MutationTaster, MutationAssessor, VEST3, GERP, phyloP, and SiPhy results, we found that the ARRB2-R8W site is highly conserved and may participate in the function disruption of the protein sequence. Therefore, we simulated the structure of the ARRB2-R8W protein. Figures 3(a) and 3(b) show images before and after the action of the wild type protein and substrate, respectively.  Figure 3(c) shows the different stages of ligand binding to the wild type protein of ARRB2. The pocket exerts three types of forces between ARRB2 and the ligand, including hydrogen bonds and hydrophobic and electrostatic forces. ASP331 binds to the ligand with the electrostatic force via a negative charge. MET207, VAL123, TRP134, PHE326, TRP334, and TYR342 bind to the ligand and form hydrophobic bonds via Van der Waals forces. ARG212, ASP331, and GLU53 bind to the ligand through conventional hydrogen bonds. We also noted a steric interaction between GLU326 and the ligand (Figure 3(d)). However, during the structural simulation, we found that the mutation at the ARRB2-R8W site had little effect on protein structure changes.

### 3.5. The ARRB2 Expression in the VPA-Induced ASD Mouse Model

**Figure 4:**
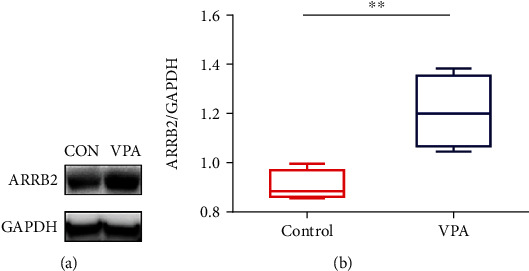
The expression of ARRB2 in the hippocampus. (a, b) Western blot analysis revealed that ARRB2 protein levels were increased in the VPA group vs. the control group. ^∗^*P* < 0.05, ^∗∗^*P* < 0.01, ^∗∗∗^*P* < 0.001. *n* = 7.

To verify our predictions, we established a VPA-induced mouse ASD model. Through behavioral testing, the mouse model was shown to exhibit autism-like behaviors such as social disorders, repetitive behaviors, and anxiety (Figure S2). Western blots of protein in the hippocampus of mice in the normal and ASD groups showed that the expression of the ARRB2 protein was increased in the ASD group (*P* < 0.01) (Figures 4(a) and 4(b)).

## 4. Conclusions

In recent years, exome sequencing has been widely used for screening of various diseases because of its simplicity and cost-effectiveness [22]. We used a series of analysis tools and found a de novo mutation of ARRB2. Mutation of ARRB2 satisfies all the screening conditions and was present in a child with ASD but not in the parents. Furthermore, mutation of this gene was predicted to be harmful by multiple software programs. The functional annotation results showed that this gene was related to neurons.

*β*-arrestin 2 is a member of the arrestin family. As a multifunctional adaptor, it plays an important role in regulating G protein-coupled receptor transport and signaling [23]. ARRB2 is highly expressed in the brain tissue and plays a key role in regulating systemic immune responses by modulating various signaling pathways [24]. Additionally, ARRB2 is associated with a variety of neurological disorders, such as Parkinson's disease [25], depressive behavior [26], and Alzheimer's disease [27]. However, few studies have revealed a direct relationship between ARRB2 and ASD. We evaluated the expression profile of ARRB2 in different human brain regions and developmental stages. Considering the high expression level of ARRB2 in the human brain, it may be important for early brain development and normal brain function, and therefore, it is a potential candidate gene for diseases related to the brain function. The hippocampus is an important brain area in the ASD research [28]. ARRB2 is expressed in the hippocampus of mice, which is consistent with previous research results by Gurevich [29]. The coexpression and genetic interaction network analysis indicated that ARRB2 may exert effects similar to several candidate ASD genes.

Considering that SNPs may cause structural or functional abnormalities of the encoded protein, tools such as MutationAssessor can be used to predict functionally harmful effects of mutation sites in protein sequences. However, in the protein structure simulation, mutation of the ARRB2-R8W site had little effect on the protein structure. The mutation site is a nonsynonymous SNP, and nonsynonymous SNPs can affect the protein function by reducing the solubility of the protein and/or the instability of the protein structure [30, 31]. R8W is highly conserved at the beginning of the amino acid sequence, which prevents it from having a major impact on the protein structure. Arginine is more hydrophobic than tryptophan, and their charges are different [32]. This change will alter the charge of the wild type residue, which may result in the loss of interaction with other molecules. Furthermore, the solubility of the protein is reduced, thereby affecting the function. Studies have hypothesized that amino acid substitutions will not cause any major structural disturbances but will only change the thermodynamic stability of the underlying state [33, 34]. In a study of the amyloid protein, the mutant fibrillation kinetics were severely slowed, and the thermodynamic stability was decreased. Despite this instability, the resulting amyloid structure was still relatively undisturbed [35].

We identified the ARRB2 gene mutation and predicted that it may be a pathological site through bioinformatic analysis. Analysis of functional mutations in individual disease-causing genes of ASD is very important to determine the mechanism and obtain pharmacological insights into ASD. Exposure to VPA can have permanent and adverse effects on the development of nerves and behavior. Children or rodents who are exposed to VPA prenatally may have a greatly increased risk of ASD [36]. To verify our predictions, we established a mouse model of ASD induced by VPA, which showed defects, including decreased social ability, increased stereotyped behavior, and anxiety (Figure S2). In this model, the ARRB2 expression was abnormal, which is consistent with our prediction.

In this study, we identified ARRB2 through WES and verified its presence through a series of bioinformatics and molecular experiments. ARRB2 may be linked to ASD. However, the mechanism by which ARRB2 is involved in ASD still requires further exploration.

## Figures and Tables

**Table 1:** Screening results.

Chr	Position (hg38)	Gene	Mutation	Transcript	Protein change	LRT	Mutation Taster	Mutation Assessor	VEST3	GERP++	phyloP46way	phyloP 100way	SiPhy_29way
chrX	70302021	KIF4A	Nonsynonymous SNV	NM_012310	p.H213P	D	D	H	0.964	5.21	1.734	8.962	13.3
chr12	57815979	AVIL	Nonsynonymous SNV	NM_006576	p.I21R	D	D	H	0.943	4.98	2.081	7.582	13.783
chr2	60945098	PUS10	Nonsynonymous SNV	NM_001322127 NM_001322123 NM_001322124 NM_144709	p.E265Q p.E488Q p.E488Q p.E488Q	D	D	M	0.948	5.67	2.836	7.445	20.142
chr5	54113362	ARL15	Nonsynonymous SNV	NM_019087	p.Q101P	D	D	M	0.935	5.9	2.254	8.04	16.325
chr17	4710743	ARRB2	Nonsynonymous SNV	NM_001257328 NM_001257329 NM_001257330 NM_001257331 NM_004313 NM_199004	p.R8W	D	D	M	0.837	5.17	1.939	5.072	9.232

LRT: (D: deleterious); MutationTaster: (D: disease_causing), both A and D indicate that the locus may be harmful; MutationAssessor: (H: high; M: medium), H and M are functional.

**Table 2:** Synapse-related genes.

Term_type	Term	GO	Number	Seq
Biological_process	Presynaptic process involved in chemical synaptic transmission	GO: 0099531	1	ENSG00000160469 (BRSK1)
Cellular_component	Synapse part	GO: 0044456	3	ENSG00000141480 (ARRB2), ENSG00000160469 (BRSK1), ENSG00000176204 (LRRTM4)
Cellular_component	Synapse	GO: 0045202	3	ENSG00000141480 (ARRB2), ENSG00000160469 (BRSK1), ENSG00000176204 (LRRTM4)

## Data Availability

The data can be seen in GSE65106 and Supplementary Materials.

## Supplementary Materials

Supplementary Materials Table S1: quality control of WES. Table S1-1: raw data results. Table S1-2: clean data results. Table. S1-3: exome coverage statistic. Table S2: indel and SNP gene. Table S2-1: indel genes. Table S2-2: SNP genes. Figure S1: GO annotation of mutant genes. Figure S2: valproic acid- (VPA-) induced mice show autism-like behavior. (a) In the three-chamber social interaction analysis, normal mice were more likely to engage in social interactions than VPA-induced mice. (b) In the elevated maze test, the percentage of time that the mice in the VPA group remained in the open arms was significantly reduced. (c) In the open field test, VPA-induced mice spent less time in the central area than control mice. (d) In the Y-maze test, mice treated with VPA showed more stereotyped repetitive behavior than control mice. Data are expressed as the mean ± SEM, ^∗^*P* < 0.05, ^∗∗^*P* < 0.01, ^∗∗∗^*P* < 0.001 vs. the control group, *n* = 10.

## References

[1] Nicolini C., Fahnestock M. (2018). The valproic acid-induced rodent model of autism. *Experimental Neurology*.

[2] Moy S. S., Nadler J. J., Magnuson T. R., Crawley J. N. (2006). Mouse models of autism spectrum disorders: the challenge for behavioral genetics. *American Journal of Medical Genetics Part C: Seminars in Medical Genetics*.

[3] Lotter V. (1966). Epidemiology of autistic conditions in young children. *Social Psychiatry and Psychiatric Epidemiology*.

[4] Fombonne E. (2009). Epidemiology of pervasive developmental disorders. *Pediatric Research*.

[5] Warrier V., Baron-Cohen S. (2019). Childhood trauma, life-time self-harm, and suicidal behaviour and ideation are associated with polygenic scores for autism. *Molecular Psychiatry*.

[6] Masi A., DeMayo M. M., Glozier N., Guastella A. J. (2017). An overview of autism spectrum disorder, heterogeneity and treatment options. *Neuroscience Bulletin*.

[7] Buescher A. V. S., Cidav Z., Knapp M., Mandell D. S. (2014). Costs of autism spectrum disorders in the United Kingdom and the United States. *JAMA Pediatrics*.

[8] Tick B., Bolton P., Happé F., Rutter M., Rijsdijk F. (2016). Heritability of autism spectrum disorders: a meta-analysis of twin studies. *Journal of Child Psychology and Psychiatry*.

[9] Gaugler T., Klei L., Sanders S. J. (2014). Most genetic risk for autism resides with common variation. *Nature Genetics*.

[10] Abrahams B. S., Arking D. E., Campbell D. B. (2013). SFARI Gene 2.0: a community-driven knowledgebase for the autism spectrum disorders (ASDs). *Molecular Autism*.

[11] Carter M. T., Scherer S. W. (2013). Autism spectrum disorder in the genetics clinic: a review. *Clinical Genetics*.

[12] Tammimies K., Marshall C. R., Walker S. (2015). Molecular diagnostic yield of chromosomal microarray analysis and whole-exome sequencing in children with autism spectrum disorder. *JAMA*.

[13] Anney R., Klei L., Pinto D. (2012). Individual common variants exert weak effects on the risk for autism spectrum disorders. *Human Molecular Genetics*.

[14] Klei L., Sanders S. J., Murtha M. T. (2012). Common genetic variants, acting additively, are a major source of risk for autism. *Molecular Autism*.

[15] Turner T. N., Coe B. P., Dickel D. E. (2017). Genomic patterns of de novo mutation in simplex autism. *Cell*.

[16] Li J., Hu S., Zhang K. (2019). A comparative study of the genetic components of three subcategories of autism spectrum disorder. *Molecular Psychiatry*.

[17] Iossifov I., O’Roak B. J., Sanders S. J. (2014). The contribution of de novo coding mutations to autism spectrum disorder. *Nature*.

[18] Krumm N., Turner T. N., Baker C. (2015). Excess of rare, inherited truncating mutations in autism. *Nature Genetics*.

[19] Lin Z., Liu Z., Li X. (2017). Whole-exome sequencing identifies a novel de novo mutation in DYNC1H1 in epileptic encephalopathies. *Scientific Reports*.

[20] St. Omer V. E. V., Ali S. F., Holson R. R., Duhart H. M., Scalzo F. M., Slikker W. (1991). Behavioral and neurochemical effects of prenatal methylenedioxymethamphetamine (MDMA) exposure in rats. *Neurotoxicology and Teratology*.

[21] Zatkova M., Bakos J., Hodosy J., Ostatnikova D. (2016). Synapse alterations in autism: review of animal model findings. *Biomedical Papers*.

[22] Sang S., Ling J., Liu X. (2019). Proband whole-exome sequencing identified genes responsible for autosomal recessive non-syndromic hearing loss in 33 chinese nuclear families. *Frontiers in Genetics*.

[23] Gupta S., Abd-Elrahman K. S., Albaker A., Dunn H. A., Ferguson S. S. G. (2019). Structural determinants governing *β*-arrestin 2 interaction with PDZ proteins and recruitment to CRFR1. *Cellular Signalling*.

[24] Zeng Y., Liang J., Weng C., Lu Z., Zhou Y. (2019). *β*-Arrestin 2 protects against neurological function defects in HSV-1-induced encephalitis mice. *Journal of Medical Virology*.

[25] Urs N. M. (2019). Methods to investigate the role of *β*-arrestin signaling in parkinson’s disease. *Beta-Arrestins*.

[26] Zhu X., Xia O., Han W. (2014). Xiao Yao San improves depressive-like behavior in rats through modulation of *β*-arrestin 2-mediated pathways in hippocampus. *Evidence-based Complementary and Alternative Medicine*.

[27] Thathiah A., Horré K., Snellinx A. (2013). *β*-arrestin 2 regulates A*β* generation and *γ*-secretase activity in Alzheimer's disease. *Nature Medicine*.

[28] Bringas M. E., Carvajal-Flores F. N., López-Ramírez T. A., Atzori M., Flores G. (2013). Rearrangement of the dendritic morphology in limbic regions and altered exploratory behavior in a rat model of autism spectrum disorder. *Neuroscience*.

[29] Gurevich E. V., Benovic J. L., Gurevich V. V. (2002). Arrestin2 and arrestin3 are differentially expressed in the rat brain during postnatal development. *Neuroscience*.

[30] Guttula P. K., Chandrasekaran G., Gupta M. K. (2019). Screening and insilico analysis of deleterious nsSNPs (missense) in human CSF3 for their effects on protein structure, stability and function. *Computational Biology and Chemistry*.

[31] Kucukkal T. G., Petukh M., Li L., Alexov E. (2015). Structural and physico-chemical effects of disease and non-disease nsSNPs on proteins. *Current Opinion in Structural Biology*.

[32] Luyten M. A., Gold M., Friesen J. D., Jones J. B. (1989). On the effects of replacing the carboxylate-binding arginine-171 by hydrophobic tyrosine or tryptophan residues in the L-lactate dehydrogenase from Bacillus stearothermophilus. *Biochemistry*.

[33] Lai J. K., Kubelka G. S., Kubelka J. (2018). Effect of mutations on the global and site-specific stability and folding of an elementary protein structural motif. *The Journal of Physical Chemistry B*.

[34] Luisi D. L., Snow C. D., Lin J.-J., Hendsch Z. S., Tidor B., Raleigh D. P. (2003). Surface salt bridges, double-mutant cycles, and protein stability: an experimental and computational analysis of the interaction of the asp 23 side chain with the N-terminus of the N-terminal domain of the ribosomal protein L9. *Biochemistry*.

[35] Adler J., Scheidt H. A., Krüger M., Thomas L., Huster D. (2014). Local interactions influence the fibrillation kinetics, structure and dynamics of A*β*(1–40) but leave the general fibril structure unchanged. *Physical Chemistry Chemical Physics*.

[36] Roullet F. I., Wollaston L., deCatanzaro D., Foster J. A. (2010). Behavioral and molecular changes in the mouse in response to prenatal exposure to the anti-epileptic drug valproic acid. *Neuroscience*.

