# Supplementary material for: Identification of a β-Arrestin 2 Mutation Related to Autism by Whole-Exome Sequencing
Source: Biomed Res Int. 2020 Nov 4;2020:8872577. doi: 10.1155/2020/8872577 (PMC7661115; doi:10.1155/2020/8872577)
Supplement: Supplementary Materials — Table S1: quality control of WES. Table S1-1: raw data results. Table S1-2: clean data results. Table. S1-3: exome coverage statistic. Table S2: indel and SNP gene. Table S2-1: indel genes. Table S2-2: SNP genes. Figure S1: GO annotation of mutant genes. Figure S2: valproic acid- (VPA-) induced mice show autism-like behavior. (a) In the three-chamber social interaction analysis, normal mice were more likely to engage in social interactions than VPA-induced mice. (b) In the elevated maze test, the percentage of time that the mice in the VPA group remained in the open arms was significantly reduced. (c) In the open field test, VPA-induced mice spent less time in the central area than control mice. (d) In the Y-maze test, mice treated with VPA showed more stereotyped repetitive behavior than control mice. Data are expressed as the mean ± SEM, ∗P < 0.05, ∗∗P < 0.01, ∗∗∗P < 0.001 vs. the control group, n = 10. [file 8872577.f1.doc]

**Supplementary Material**

**Table. S1 Quality Control of WES**

**Table. S1-1 Rawdata Results**

| Sample_ID | Total_Reads | Total_Bases | Error% | Q20% | Q30% | GC% |
| --- | --- | --- | --- | --- | --- | --- |
| ertong | 74426528 | 11163979200 | 0.0271 | 97.19 | 92.18 | 49.24 |
| fu | 79967000 | 11995050000 | 0.0278 | 96.88 | 91.61 | 48.89 |
| mu | 80879254 | 12131888100 | 0.0266 | 97.43 | 92.58 | 49 |

**Table. S1-2 Cleandata Results**

| Sample_ID | Total_Reads | Total_Bases | Error% | Q20% | Q30% | GC% |
| --- | --- | --- | --- | --- | --- | --- |
| ertong | 73111446 | 10871193211 | 0.0263 | 97.68 | 92.76 | 48.69 |
| fu | 77729416 | 11538368807 | 0.0265 | 97.6 | 92.51 | 47.96 |
| mu | 79664510 | 11803191762 | 0.0258 | 97.86 | 93.14 | 48.5 |

**Table. S1-3 Exome Coverage Statistic**

| Sample | Exome Size (bp) | Covered Bases (bp) | Exome Coverage | Exome Coverage (depth>=5) | Exome Coverage (depth>=20) | Exome Mapped Data (Mb) | Exome Average Depth (x) |
| --- | --- | --- | --- | --- | --- | --- | --- |
| ertong | 60700153 | 60342140 | 99.41 | 97.85 | 75.85 | 2403.18 | 39.83 |
| fu | 60700153 | 60333766 | 99.4 | 97.91 | 79.74 | 2519.93 | 41.77 |
| mu | 60700153 | 60207470 | 99.19 | 97.72 | 78.79 | 2568.42 | 42.66 |

**Table. S2 Indel and SNP gene**

**Table. S2-1 Indel Genes**

| Chr | Start | End | Ref | Alt | Gene |
| --- | --- | --- | --- | --- | --- |
| chrX | 136879427 | 136879428 | TG | - | RBMX |
| chr19 | 16082045 | 16082045 | - | CC | TPM4 |
| chr1 | 200409604 | 200409606 | GCC | - | ZNF281 |
| chr6 | 44002767 | 44002769 | GCG | - | C6orf223 |
| chr2 | 174349047 | 174349049 | GAG | - | CIR1 |
| chr7 | 87553903 | 87553903 | - | AT | ABCB1 |
| chr1 | 182952866 | 182952868 | CCG | - | SHCBP1L |
| chr14 | 24300643 | 24300643 | - | GAGGAG | NOP9 |
| chr1 | 8324504 | 8324504 | - | G | SLC45A1 |
| chr3 | 75738116 | 75738117 | CT | - | ZNF717 |
| chr20 | 326558 | 326560 | GCC | - | SOX12 |
| chr12 | 80254523 | 80254524 | GT | - | OTOGL |
| chr10 | 46287305 | 46287307 | CTG | - | ANTXRL |
| chr7 | 87553901 | 87553902 | TT | - | ABCB1 |
| chr17 | 40818851 | 40818851 | - | GCTGCCGCCGCCGTATCCGCCGCCGGAGCT | KRT10 |
| chr7 | 139409619 | 139409620 | AG | - | C7orf55-LUC7L2;LUC7L2 |
| chr2 | 171692828 | 171692828 | - | A | DYNC1I2 |
| chr6 | 156778293 | 156778295 | CAG | - | ARID1B |
| chr19 | 932497 | 932499 | GAG | - | ARID3A |
| chrX | 16832726 | 16832727 | AG | - | TXLNG |

**Table. S2-2 SNP Genes**

| Chr | Start | End | Ref | Alt | Gene |
| --- | --- | --- | --- | --- | --- |
| chr1 | 109189660 | 109189660 | A | C | KIAA1324 |
| chr19 | 6693445 | 6693445 | A | T | C3 |
| chr10 | 32453369 | 32453369 | A | G | CCDC7 |
| chr15 | 45689171 | 45689171 | A | C | SQOR |
| chrX | 70302021 | 70302021 | A | C | KIF4A |
| chr3 | 42557651 | 42557651 | G | C | SEC22C |
| chr17 | 75504916 | 75504916 | A | T | CASKIN2 |
| chr19 | 55092658 | 55092658 | T | A | PPP1R12C |
| chr13 | 25096769 | 25096769 | C | A | PABPC3 |
| chr14 | 104951898 | 104951898 | A | T | AHNAK2 |
| chr2 | 187467823 | 187467823 | C | G | TFPI |
| chr3 | 75737051 | 75737051 | T | C | ZNF717 |
| chr22 | 38689095 | 38689095 | C | T | JOSD1 |
| chr2 | 99379368 | 99379368 | A | C | EIF5B |
| chr13 | 25096739 | 25096739 | G | A | PABPC3 |
| chr3 | 75738148 | 75738148 | G | A | ZNF717 |
| chr9 | 17143348 | 17143348 | A | C | CNTLN |
| chr1 | 6249964 | 6249964 | C | T | GPR153 |
| chr3 | 113661195 | 113661195 | T | G | USF3 |
| chr16 | 29383028 | 29383028 | C | T | NPIPB11 |
| chr2 | 219567079 | 219567079 | C | T | OBSL1 |
| chr7 | 95311470 | 95311470 | T | C | PON1 |
| chr20 | 49249611 | 49249611 | A | G | ZNFX1 |
| chr2 | 77519101 | 77519101 | G | C | LRRTM4 |
| chr16 | 29383050 | 29383050 | T | C | NPIPB11 |
| chr9 | 137069995 | 137069995 | G | C | SAPCD2 |
| chr2 | 28538345 | 28538345 | T | G | PLB1 |
| chr9 | 114350911 | 114350911 | A | T | AKNA |
| chr5 | 83204915 | 83204915 | G | T | XRCC4 |
| chr12 | 5994567 | 5994567 | C | T | VWF |
| chr12 | 27687404 | 27687404 | A | T | PPFIBP1 |
| chr3 | 75737348 | 75737348 | C | T | ZNF717 |
| chr3 | 75737365 | 75737365 | G | T | ZNF717 |
| chr17 | 63238053 | 63238053 | A | C | TANC2 |
| chr11 | 64836175 | 64836175 | G | T | CDC42BPG |
| chr17 | 81511394 | 81511394 | C | G | ACTG1 |
| chr9 | 114350916 | 114350916 | A | T | AKNA |
| chr13 | 25096781 | 25096781 | A | G | PABPC3 |
| chr2 | 187467845 | 187467845 | A | G | TFPI |
| chr5 | 160119339 | 160119339 | T | A | PWWP2A |
| chrX | 41337415 | 41337415 | G | A | DDX3X |
| chr3 | 75738118 | 75738118 | G | A | ZNF717 |
| chr1 | 19657029 | 19657029 | A | C | MINOS1-NBL1;NBL1 |
| chr19 | 6693463 | 6693463 | C | T | C3 |
| chr2 | 187467818 | 187467818 | C | G | TFPI |
| chr3 | 75738128 | 75738128 | A | T | ZNF717 |
| chr14 | 64568349 | 64568349 | G | T | PPP1R36 |
| chr19 | 55304750 | 55304750 | C | T | BRSK1 |
| chr3 | 75737791 | 75737791 | C | A | ZNF717 |
| chr19 | 41128072 | 41128072 | G | A | CYP2F1 |
| chr19 | 2275870 | 2275870 | G | A | C19orf35 |
| chr16 | 51141959 | 51141959 | C | T | SALL1 |
| chr11 | 122981548 | 122981548 | C | A | BSX |
| chr21 | 44261226 | 44261226 | C | T | DNMT3L |
| chr3 | 75738137 | 75738137 | T | C | ZNF717 |
| chr2 | 187467830 | 187467830 | G | C | TFPI |
| chr2 | 187467848 | 187467848 | A | G | TFPI |
| chr14 | 104951897 | 104951897 | G | A | AHNAK2 |
| chr5 | 54113362 | 54113362 | T | G | ARL15 |
| chr2 | 187467833 | 187467833 | C | T | TFPI |
| chr17 | 4710743 | 4710743 | A | T | ARRB2 |
| chr1 | 166860285 | 166860285 | C | T | TADA1 |
| chr6 | 127516120 | 127516120 | T | A | SOGA3 |
| chr1 | 19087876 | 19087876 | T | G | UBR4 |
| chr3 | 75738151 | 75738151 | T | C | ZNF717 |
| chr3 | 75737302 | 75737302 | C | T | ZNF717 |
| chr19 | 17286694 | 17286694 | G | T | ANKLE1 |
| chr15 | 63043746 | 63043746 | T | C | TPM1 |
| chr7 | 100966916 | 100966916 | T | A | MUC3A |
| chr7 | 150803446 | 150803446 | G | A | TMEM176A |
| chr3 | 130567187 | 130567187 | G | C | COL6A6 |
| chr1 | 119626031 | 119626031 | A | C | ZNF697 |
| chr19 | 55092655 | 55092655 | T | A | PPP1R12C |
| chr12 | 57815979 | 57815979 | A | C | AVIL |
| chrX | 154931153 | 154931153 | A | C | F8 |
| chr1 | 160155126 | 160155126 | T | A | ATP1A4 |
| chr12 | 14553243 | 14553243 | A | C | PLBD1 |
| chr21 | 10605542 | 10605542 | G | A | TPTE |
| chr1 | 248039172 | 248039172 | A | G | OR2L2 |
| chr3 | 75741362 | 75741362 | T | C | ZNF717 |
| chr3 | 75738859 | 75738859 | A | C | ZNF717 |
| chr12 | 109913013 | 109913013 | G | A | TCHP |
| chr15 | 62067843 | 62067843 | G | A | C2CD4A |
| chr2 | 132732036 | 132732036 | A | C | NCKAP5 |
| chr3 | 48420209 | 48420209 | A | T | PLXNB1 |
| chr11 | 71816850 | 71816850 | G | A | ZNF705E |
| chr2 | 60945098 | 60945098 | C | G | PUS10 |
| chr7 | 6654579 | 6654579 | C | A | ZNF316 |

**
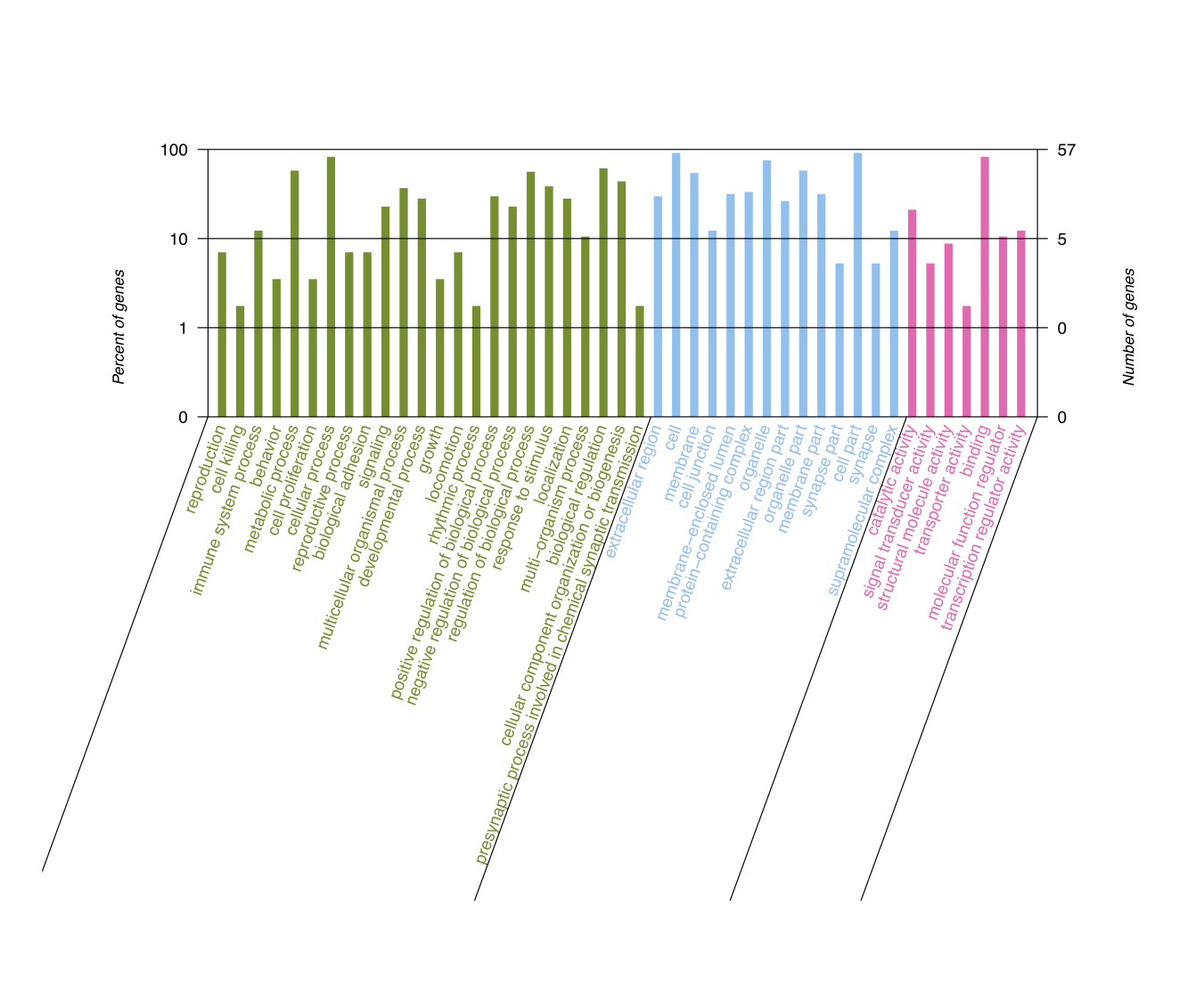
**

**Fig. S1** GO annotation of mutant genes.

**2.5 Behavioral tests**

**2.5.1 Three-chamber social test**

The device consisted of two side chambers, a central chamber, and two identical transparent cylinders (60 (length) × 40 (width) × 30 (height) cm)^[1]^. The three chambers were interconnected. During the adaptation phase, two empty cylinders were placed in the side chambers, and the mice were placed in the central chamber. Their behavior in the three chambers was recorded for 10 minutes. During the social ability test phase, an unfamiliar C57 mouse (Stranger 1) was placed in the left chamber, and an empty cylinder was still placed in the right chamber. Ethovision was used to automatically calculate the time spent in each area.

**2.5.2 Y maze**

Repetitive stereotypes are an important feature of ASD. The Y maze device (25 cm × 8 cm × 15 cm) was used to study the repetitive behavior of mice^[2]^. The Y maze device consists of three identical arms. Mice were placed in the starting arm of the Y-maze and allowed to explore randomly for 10 minutes. The spontaneous alternation rate was recorded.

% Spontaneous alternations = (Total alternations / Total arms entered − 2) × 100

**2.5.3 Open field test**

The open field test assesses the mood of mice^[3]^. In this test, a box with a length × width × height of 40 × 40 × 50 cm was used. The bottom of the box was divided into 16 equal squares. The four small squares in the central area were designated as the central active area. Ethovision automatically tracked and analyzed the mouse's activity time in the central area within 10 minutes, and the anxiety of the mouse was observed.

**2.5.4 Elevated plus maze test**

The elevated plus maze assesses anxiety in animals^[4]^ because they tend to explore open arms rather than closed arms. The device includes two open arms, two closed arms, and a common central platform 40 cm above the ground. The mouse is lowered from the central platform while facing the open arm. The time that the mouse remained in each arm was observed and recorded for 5 minutes.


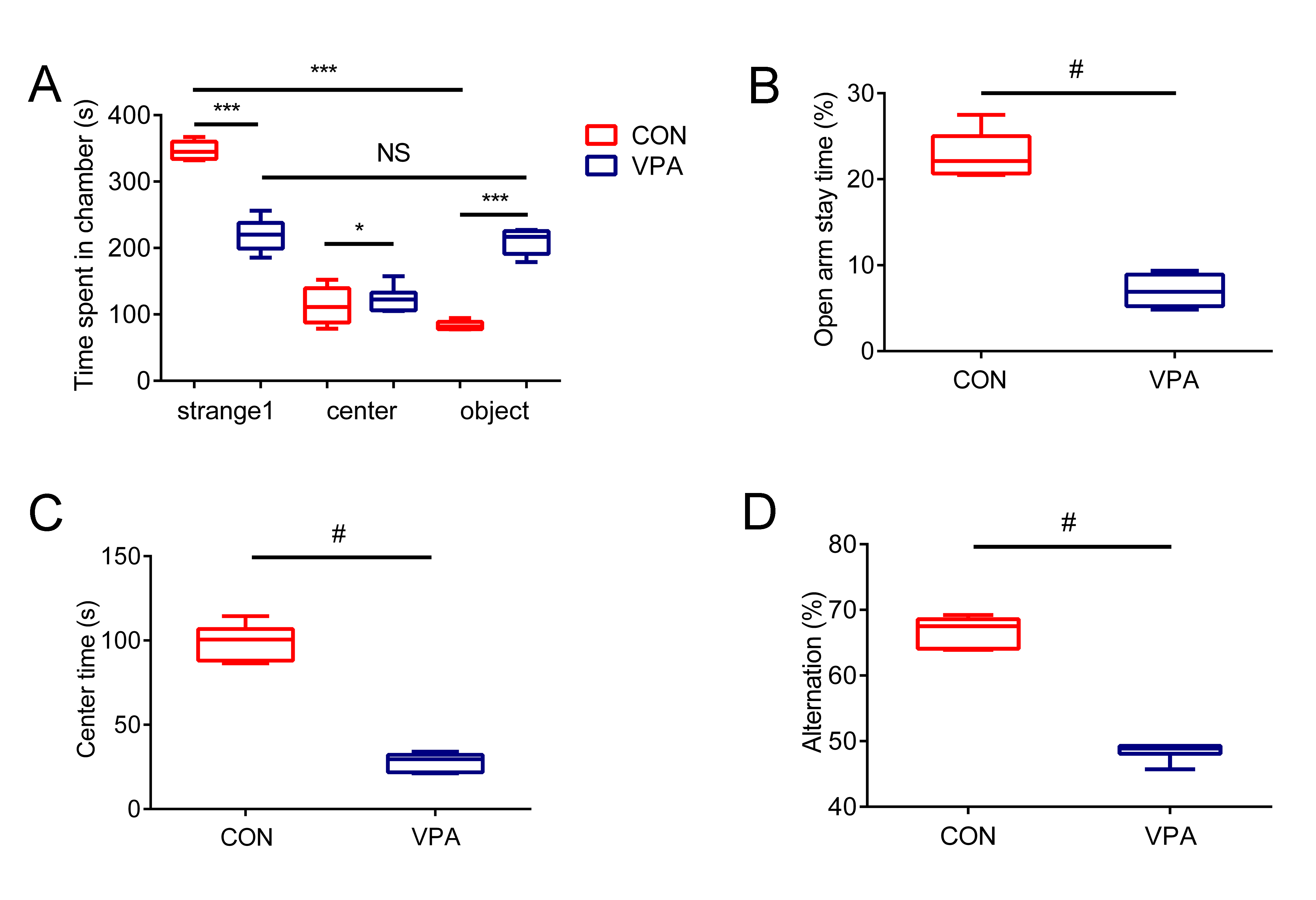


**Fig. S2** V[alproic acid](http://www.baidu.com/link?url=qEbojurlu0j1dAGRdb0DTJ0TKKXOAqgO6eFysiPgqNNQV9fMAUg-36ithfLsozy7kQLbiwAMJwpe3i1aKNTT2q) (VPA)-induced mice show autism-like behavior. (A) In the three-chamber social interaction analysis, normal mice were more likely to engage in social interactions than VPA-induced mice. (B) In the elevated maze test, the percentage of time that the mice in the VPA group remained in the open arms was significantly reduced. (C) In the open field test, VPA-induced mice spent less time in the central area than control mice. (D) In the Y-maze test, mice treated with VPA showed more stereotyped repetitive behavior than control mice. Data are expressed as the mean ± SEM, *P < 0.05, **P < 0.01, ***P < 0.001 vs. the control group, n = 10.

**References**

1. M. Yang, J. L. Silverman,J. N. Crawley. Automated three-chambered social approach task for mice. Curr. Protoc. Neurosci., 56 (2011), <https://doi.org/10.1002/0471142301.ns0826s56>.
2. S. M. Hölter, L. Garrett, J. Einicke, et al. Assessing cognition in mice. Curr. Protoc. Mouse Biol., 5 (2015), pp. 331-358, <https://doi.org/10.1002/9780470942390.mo150068>.
3. T. Wong, I. Bestardlorigados,D. A. Crawford. Autism-related behaviors in the cyclooxygenase-2-deficient mouse model. Genes Brain and Behavior, 18 (2019), p. e12506, <https://doi.org/10.1111/gbb.12506>.
4. S. A. Norton, J. J. Gifford, A. P. Pawlak, et al. Long-lasting behavioral and neuroanatomical effects of postnatal valproic acid treatment. Neuroscience, (2020), pp. 8-21, <https://doi.org/10.1016/j.neuroscience.2020.02.029>.
